# Challenges and stresses experienced by athletes and coaches leading up to the Paralympic Games

**DOI:** 10.1371/journal.pone.0251171

**Published:** 2021-05-06

**Authors:** N. Dehghansai, R. A. Pinder, J. Baker, I. Renshaw

**Affiliations:** 1 School of Kinesiology and Health Science, York University, Toronto, Canada; 2 Paralympic Innovation, Paralympics Australia, Adelaide, Australia; 3 School of Exercise and Nutrition Sciences, Queensland University of Technology, Brisbane, Australia; University of Pittsburgh, UNITED STATES

## Abstract

The demands of high-performance sport are exacerbated during the lead up to the Major Games (i.e., Paralympics). The purpose of this study was to better understand the challenges experienced and strategies utilized by Australian athletes (n = 7) and coaches (n = 5) preparing for the Tokyo Paralympic Games using semi-structured interviews. The thematic analysis highlighted challenges specific to participants’ sport (e.g., budgetary constraints, decentralized experiences, athletes with various impairments), personal life (e.g., moving cities to access coaching, postponing vocational/educational developments, isolation from social circles), and associated uncertainties (e.g., COVID-19, qualifications, accreditations). Participants managed these challenges by utilizing strategies to ‘anticipate and prepare’ (e.g., detailed planning, effective communication, contingency plans) and ‘manage expectations’ (e.g., understanding specific roles and boundaries, focusing on the process [i.e., effort over results]). Trust and communication between athletes and coaches was key in coaches’ better understanding of how athletes’ impairments interact with their training and competition environments and tailor support to each athlete’s unique needs. Last, participants reflected on the ‘pressure’ of the Games due to their performance having an impact on their career trajectory ‘post-Tokyo’ with some athletes contemplating retirement and others realizing the consequences of their performance on sport-related vocation and sponsorship. Coaches also accepted the success of their programs and job security will depend on outcomes at the Games. The findings from this study shed light on factors to consider to reduce challenges for teams preparing for major competitions but also highlight key practical implications to support athletes and coaches leading up, during, and post-major Games.

## Introduction

While the benefits of sport participation such as the development of motor skills and improved physical and psychosocial health are widely reported [1–3], the stresses and challenges associated with high-performance sport may buffer these positive effects [4]. Due to high-expectations, high-intensity training environments, increased media exposure, and more severe consequences for below-average performances, elite athletes are exposed to exhaustion and burn out, prone to physical injuries, and at the extreme cases, psychological and physical disorders [5, 6]. These challenges are especially difficult for athletes emerging onto the national and international competition, from both athletic (keeping up with performance expectations) and social development (fitting in and relationship with others; [4]) perspectives.

In elite and/or professional contexts, athletes are not alone in dealing with a stressful environment. Similarly, coaches are often faced with lofty expectations to support athletes’ potential at international events [7]. While there is research examining the stress coaches feel and the strategies they use to cope with this stress [8, 9], there has been little work focusing on coaches’ experiences and how they deal with the increasing challenges prevalent in high-performance settings [7, 10]. The continuous demands to support athletes, balancing expectations of administrative stakeholders, parents, and the athletes along with the increased scrutiny by media and news outlets can result in coaches feeling exhausted and burned out [8, 11, 12] Meanwhile, coaches with high-workloads and low self-regulatory skills have reported experiencing higher levels of exhaustion throughout the season [8, 11, 12]. One of the ways coaches have reported dealing with these demands is by remaining adaptive and learning ‘on the job’, discussing opportunities with others, and relying on their prior experience as athletes [13]. Considering the complexity of the coaching process and the demands placed on coaches from various sources, it is vital to develop a better understanding of the challenges and coping mechanisms coaches utilize to ensure their own, and their athletes’ well-being [14].

Challenges for coaches and athletes in high-performance settings can be exacerbated leading up to major Games (e.g., World Championships, Olympics, or Paralympics). Understandably, as the intensity of training and the number of qualification tournaments and training camps increases, life outside of sport is usually put on hold while within sport demands increase (i.e., media engagements, travel, etc.; [15–17]). In addition, high-performance coaches typically receive Quadrennial contracts and coach/athlete performance expectations increase the implications and consequences of the team’s performance at the Games.

### The Paralympic context

While there is some literature on these changes and their implications for athletes and coaches participating in able-bodied sport [15–18], there has been very little exploration of these issues in Paralympic contexts [19, 20]. Arguably, there are similarities between the two sporting Games (i.e., Quadrennial format and peak event for most competing athletes and their coaches, intense environments, increased media exposure, and prestigious accolades [21–23]; however, it is important to consider that there may be challenges that are unique to Paralympic sport contexts. For example, there are similarities between able-bodied and Para sport coaching (i.e., the value of using autonomy-supportive strategies), but there are also aspects when coaching elite athletes with disabilities that require a more adaptive approach (i.e., tailoring training to athletes’ unique abilities or dealing with social stigmas in public settings; [20, 24–30]. In addition, there is considerable literature noting how Paralympic coaches need to adapt and utilize different methods of learning (i.e., formal, nonformal, informal) to combat the lack of resources and support available to them [31–34]. However, we have limited understanding of the challenges Paralympic coaches face preparing athletes for the Paralympic Games and the strategies they utilize to combat these challenges.

Similarly, there is also limited knowledge of the challenges Paralympic athletes face in preparation for these types of Major Games. In studying motivational profiles of Paralympic and Olympic athletes, Pensgaard and colleagues [35] concluded both groups have similar motivation profiles. However, due to their impairment or injury-related trauma, Paralympic athletes use different coping strategies (i.e., more mastery-oriented climate) while reflecting different mood profiles and satisfactory responses (i.e., significantly more satisfied with effort and results), suggesting Paralympic athletes cope with stressors in their environment differently. Both Campbell and Jones [36, 37] and Jefferies and colleagues [38] have explored the associated stress of Paralympic athletes before, during, and post-competition, with athletes worrying about their contribution to their team, the adequacy of their preparation for the games (e.g., whether training would result in match performance, not feeling fully fit, appropriate pre-match preparation), and how they will manage psychosocial pressures (preventing or treating nagging injuries, personal, and team performance potentials, consequences of results). Team culture (i.e., teammate personalities, conflicting goals, teammate behavior and interactions, roles and responsibilities, team disparity) was also identified as a key contributor to athlete stress and anger, leading to elevated stress levels in the build-up to competition [2, 3]. Given our limited understanding in this area and (potentially) unique challenges (e.g., disability-related nuances, lack of resources or support, etc.) experienced by Paralympic athletes and coaches in the lead-up to a major Games, the purpose of this project was to explore the challenges, solutions, and strategies utilized by athletes and coaches in the lead up to the Tokyo 2020 Paralympic Games.

## Method

### Participants

#### Recruitment

Members of two Paralympic teams in Australia were contacted by Paralympics Australia (note: the sports remain anonymous to protect participant identity). After sport approval, six coaches and eight athletes were contacted via email with a letter explaining project details. Five coaches and seven athletes agreed to participate. Interviews were held in person with all but two athletes, which were recorded during a video call using Zoom (Zoom Video Communications, Inc.). Based on the responses, the authors felt data saturation was reached and no further recruitment was required. Interviews ranged between 25–60 minutes in duration. The interview structure was designed to allow participants to share their experiences and some expressed their opinions in more detail, thus, the discrepancy between interview durations. Interviews were held from January 05, 2020, to February 05, 2020. It is important to note that this period was before the COVID-19 (a severe acute respiratory syndrome coronavirus 2 (SARS-CoV-2), first identified in December 2019 in Wuhan, China and officially declared a worldwide pandemic by World Health Organization on March 11, 2020 [39]) outbreak was declared a pandemic by the World Health Organization [40]. This study took place prior to the postponement of the 2020 Games as a result of the COVID-19 global pandemic. All data collection for this project was completed with the expectation of these Games going ahead in summer 2020.

#### Background

All participants reported previous experience at international events. Three of five coaches had coached at a Paralympic Games previously, while five of seven athletes reported competing at the previous Paralympic Games in Rio de Janeiro in 2016. Table 1 provides basic details of all participants sporting careers; this information is intentionally limited to retain the anonymity of participants due to the small pool of Australian Paralympic athletes and coaches.

**Table 1:** Details for each participant in the interview.

Coaches*	Years in Current Role	Athletes	Highest competitive accomplishment
C1	Four	A1	World Champion Medalist
C2	Eleven	A2	World Champion Medalist and Paralympian
C3	Four	A3	World Champion Medalist
C4	Four	A4	World Champion and Paralympic Medalist
C5	Twelve	A5	World Champion and Paralympic Medalist
		A6	World Champion and Paralympic Medalist
		A7	Paralympic Medalist

*All the coaches reported having more than a decade of experience coaching with experience ranging from Olympic to Paralympic teams.

**All but one athlete had already qualified for the Tokyo 2020 Paralympic games.

#### Ethical considerations

Ethics was obtained from Human Participants—Research & Innovation (HPRC) at York University with permission to collect the data pertaining to Australian participants. Participants provided informed consent form before the start of each interview. The authors remained cognizant of the role of Paralympics Australia (PA) from a relational ethics lens (i.e., the relationship between PA and sports may influence the interview process and how the interviews are interpreted; [41]). However, the lead author, who conducted the interviews and facilitated the thematic analysis had a limited relationship with the athletes and coaches and was not directly affiliated with PA, thus, reducing concerns regarding relational ethics and bias towards existing relationships [42–44]. The final report was shared with the participants to allow for any clarification or retraction of statements. This process did not result in any changes. The authors also provided participants with the opportunity to review their transcripts but none of the participants requested a copy. Subsequently, the current manuscript in its original form was shared with participants prior to submission for publication.

### Methodology

Semi-structured interviews are commonly used in research focusing on better understanding the experience of athletes and coaches [10, 45]. While the researcher has a general set of guiding questions to lead the interview process, the semi-structured nature allows the participants to introduce new ideas and share experiences through their narrative. We chose this process to enable a deeper understanding of athletes’ and coaches’ experiences, challenges, and strategies in relation to preparing for a Paralympic Games.

#### Philosophical assumption

This study was grounded ontologically in relativism (i.e., multiple realities exist which are subjective and mentally constructed) and epistemologically in constructivism (i.e., knowledge is constructed based on individual’s interactions and experiences in a specific context [46, 47]). Each athlete and coach experienced the process of preparation for the Games differently, thus it is vital to better understand the relationship of the individual within their current system from the lens of each person’s narrative. Thus, the narrative constructivism allows us to address the objective of this research by capturing the meanings associated with each person’s story and how it coordinates with others’ experiences in their sporting system [45, 48]. It is important to consider each participant’s narrative as more than merely a story-telling opportunity as their experiences are part of the larger Paralympic sporting system [49, 50]. Thus, understanding these narratives help understand the larger system in which each participant resides.

#### Methodological rigor

Consistent with an ontological relativism and epistemological constructivism approach, a guiding list of criteria was set out to establish the rigor of this study design [47, 51]. The *worthiness of topic* was demonstrated from the limited literature that exists in this field that explores athlete and coach challenges leading up to the Paralympic Games. Authors aligned their methods with previous literature to ensure a *rich rigor* process for the interview procedure, data exploration and theme generation. In addition, the authors utilized the technique of ‘critical friend’ among the lead and co-authors to use each other as a ‘theoretical sounding board’ to evaluate, interpret, and develop themes throughout the project [52]. Throughout this process, the lead author was continuously probed to ensure his bias and personal perspectives did not dilute the exploration process and compromise the content.

#### Procedure and interview guide

A series of general topics were approved via a discussion between the authors and probe questions were implemented to keep consistent with the topic and elicit open discussion [53]. The interview guide was then shared with an external committee within Paralympic Australia to review and provide feedback. This committee oversaw athlete wellness and had athlete representatives as members who reviewed and provided their approval on the content of the guide. The interview guideline was organized into four main sections: 1) challenges leading up to the Games, 2) expectations of themselves, their athletes or coaches, and their staff at the Games, 3) uncertainties on their preparation, the Games itself, and transition after the Games, and 4) closing questions.

### Data analysis

Recordings of the interviews were transcribed verbatim and data exploration was guided through the six-phased inductive steps of thematic analysis [54]. These steps included a familiarity of the content by re-reading the transcripts, using NVivo (NVivo qualitative analysis software: Version 12), and noting significant thoughts and patterns. These codes were then refined and grouped into higher- and sub-themes. Across the five levels of coding, re-evaluation and critical discussions by other authors [47], overlapping themes were merged, some themes were dissolved and a final structure of three higher-themes and seven sub-themes remained (refer to Table 2 for details of the theme breakdown).

**Table 2:** Theme breakdown.

Theme	Sub-Theme	Examples
‘Challenges’ Current or anticipated challenges leading up to the Games	Sport-Specific Challenges specific to the participants’ sport with direct implications	• Budgetary constraints • Decentralized programs • Remote coaching • Tailored training programs • Impairment-variations • Team-cohesion
	Personal Challenges with direct impact on participants’ personal lives	• Lack of family time • Moving houses, cities, countries • Limited opportunity for vocation
	Uncertainties Inconclusive scenarios with the potential to play a role in development and preparation for the Games	• COVID-19 • Qualifications • Accreditation
Strategies Some of the strategies currently implemented to reduce the impact or prevent challenges from (re)occurring	Anticipation and Preparation The anticipation of certain obstacles/outcomes and developing contingency plans or communication methods to reduce the impact of challenges	• Communication • Detailed Planning • Contingency Plans • Previous Games’ Experience
	Managing Expectations A better understanding of each other and specific roles to reduce miscommunication, expectations, and misunderstanding	• Knowing each other’s role • Knowing each other’s boundaries • Process over outcome
Post-Tokyo Participants’ thoughts, ideas, and plans for post-Games. Mainly hinged on performance outcomes during the Games	Pressure The extensive build-up and opportunities that are dependent on performance at the Games creates additional pressure	• Job on the line • Performance = Career opportunities • Performance = Retirement
	Personal The extensive and increase commitment leading up to the Games needs to be balanced with a break to focus on other aspects of life that were put on pause	• Reconnect with social circles • Education and vocation • Mental and physical break

## Results and discussion

Each of the overarching themes contained sub-themes that captured nuances regarding participants’ experiences, challenges, and strategies to deal with current or anticipated challenges. The three overarching themes were ‘challenges,’ ‘strategies,’ and ‘post-Tokyo.’ The theme ‘challenges’ captured some of the shortcomings athletes and coaches currently face or anticipate might come up in the near future. The sub-themes for challenges were ‘sport-specific,’ ‘personal,’ and ‘uncertainties.’ The strategies examined ways participants mitigated some of the challenges and methods used to either reduce or eliminate challenges. The sub-themes of this general theme were ‘anticipation and preparation’ and ‘managing expectations.’ Post-Tokyo consisted of athletes and coaches’ prediction of events based on the outcomes at the Games and consisted of the sub-themes ‘pressure’ and ‘personal.’

### Challenges

Both athletes and coaches highlighted challenges that were organized into sport-specific, personal, and uncertainties. Sport-specific challenges captured the experience of athletes and coaches directly within the sporting environment. These challenges were either specific to their sport (e.g., budgetary constraints), tied directly to individuals (e.g., moving cities to access coaches), or factors that occurred within the sporting environments (e.g., training and competition contexts). Challenges that were rooted within the sport were organized into the sport-specific category while sport-related challenges that were specific to the individual and their circumstances were organized into the personal theme. Due to the uniqueness and depth of issues associated with uncertainties, these experiences were captured under the uncertainties theme to demonstrate the vastness of the theme that emerged during the interviews.

#### Sport-specific

While athletes and coaches expressed similar sport-specific challenges, participants’ experiences and their perspectives of how these challenges impacted them varied. For example, coaches highlighted working with budgetary constraints as a challenge because as the Paralympic Games near, there are qualifying tournaments that athletes need to attend, coupled with opportunities for pathway athletes (i.e., athletes currently in the sporting pathway that have not qualified for the upcoming games; however, have displayed potential to emerge as future Paralympic athletes) to gain experience. However, limited budgets from senior management coupled with high expectations for gold medal targets forced coaches and directors to make tough decisions regarding who could attend these tournaments. As alluded to by C1:

We have a limited budget … [and] to do what we wanted to, but also as [our sport] and para-sports evolving, we’ve had to make some pretty tough decisions on narrowing our focus on athletes. And so, athletes are missing out and so that’s been quite a big shift nearly last year’s [competition] in 2019, we took 15 athletes, this year we took eight.

These challenges can be exacerbated because of the need to travel for high-quality opposition given Australia’s geographical location relative to other countries. The constant travel was highlighted as a challenge by the athletes due to the physical and psychological demands; changing time zones and accommodation (i.e., sleeping away from home), as well as the stress of flying which contributed to lack of sleep, slower recovery, and reduced energy. As highlighted by A3, traveling on long-flights impacted her training, recovery, and performance in the past:

Just being able to accept it and know that we’ll have the right strategies for Tokyo, hopefully, it’ll be good. We went to Canada, with time zone, it was bad, so [with Tokyo] the time zone isn’t too bad.

Australia’s geography is a further challenge, especially for sports with limited budgets. Unfortunately, being decentralized (train locally and only meeting with national team [coaches and teammates] during tournaments and training camps) in a country like Australia requires frequent travel for athletes to maintain a connection with coaches who can live significant distances away even when they live in the same state (e.g., an athlete living in Cairns in Queensland whose coach lives in the capital city of Queensland, Brisbane are 1,700 km apart and would need to take an 18-hour car journey or a 2:20 flight to meet face-to-face), a point often alluded to by both athletes and coaches.

The incurred cost, along with preparation and planning for domestic and international travel, is a common challenge Paralympic coaches have to deal with [20, 55]. Interestingly, the commonly reported challenges associated with athletes’ experience during travel, especially in dealings with airlines and freight carriers (i.e., boarding, bathroom access, luggage, and wheelchair accommodation [20]) was not reported as an issue by coaches or athletes in this study. Athletes only reflected on the impact of travel due to their impairment, which led to fatigue and negatively impacted their training/competition performance. As highlighted by A1:

I travel really poorly, which is why [last competition]… I felt so bad on the day. I think is what we did last year [that worked], we had like a training camp in [another country]. I went three weeks before everyone else left here. And that was awesome because it gave me like all of that time to be not under stress, trying to recover from travel.

From a psychological standpoint, being away from family, friends, and one’s daily routine adds to the physical stress, leaving little time for recovery. This could be reflected through subtle things such as sleeping in their own beds, as alluded to by A2, “And I sleep in my own bed and can still walk my dog. So those things in life.” The lack of challenges previously reported in the literature could be twofold: first, the group of athletes and coaches in this study were all competing at the elite level and perhaps more accustomed to the routines associated with traveling. Second, it has been suggested in the literature that Paralympic athletes represent the less severe impairments within the disability community [56]. and this cohort of athletes may have not been exposed to similar experiences reported previously in the literature.

From a coaching perspective, understanding athletes’ abilities, tendencies, and weaknesses are paramount to designing a training environment conducive to their athlete [24]. However, having finite resources and therefore limited time with athletes, makes this a challenge. Proximity helps develop a trust that underpins working together, and this trust is the basis for effective coaching. One of the coaches, C1 highlighted how building of trust evolves slowly and opportunities must be taken wherever possible, including camps and traveling for tournaments:

This last year their first trip away to Europe, it was pretty much a new coaching team, and it took a fair bit to get the athletes to have the bond and to trust us as the coaches. So, I think that was a big change for the athletes. It’s getting better and better from each trip away as a group. And yeah, and that’s coaching in general though. You’ve got to build that relationship with the athletes, so it does take time.

Athletes also expressed the importance of being comfortable with their coaches so that they can express their training preferences and tendencies as well as share personal information about their impairment, so the coaches have a better understanding of athletes’ function to adjust training and recovery accordingly. This was highlighted by A3:

Maybe just communicating of if I’m not feeling the greatest. I haven’t pulled up as great from the session before. Because it’s been really good, but for me, being able to be honest even though I want to try and complete the full training session. Yeah, just communicating really and making sure I know limits.

And it was further expanded on by A1:

So I think that’s been a challenge for my coaches and me to sort of know when I have to speak up and be really certain about it, like, "I don’t think I can do that," or, "I don’t think I should do that." I don’t do that enough because it’s really… I find it really difficult to talk about, and I don’t want to acknowledge when things aren’t going well.

The decentralized environment also restricts bonding opportunities for squad members largely to camps and tournaments. Coaches expressed the importance of utilizing opportunities during camps to develop group chemistry, as they were aware that the Paralympics environment will be stressful and tense. Therefore, ensuring athletes understand each other’s tendencies and how each prefers to prepare for competition, or responds in different situations (e.g. following a loss) would be paramount to reducing issues on competition day. As alluded to by Banack and colleagues [24], the psychological needs of Paralympic athletes can vary by context and coaches need to consider athlete’s attributes (gender, impairment, most recent competition performance, living conditions) in relating and bonding with athletes.

Some of the participants also noted the pressure of performing at the Games and its impact on current training. Both athletes and coaches highlighted the importance of strategic training (i.e., planning ahead and training smart), but also the need to focus on appropriate recovery. The elevated stress of upcoming competition is a commonly reported experience by Paralympic athletes and similar to prior reports, athletes best cope with the pressure by utilizing problem-focused coping mechanisms including smart training and recovery planning [3]. Experienced athletes (which included a wide range of impairment types) highlighted the need to train and recover strategically to reduce the chances of overload on their bodies. The long-term impact of impairment and the need to identify triggers and minimize overloading in training was highlighted by A3:

It was good to get back into [training], but it was a little bit hard just because I seem to not be able to control it a bit. I’m on medication but it doesn’t seem to always be effective … It’s kind of an ongoing challenge just being able to like, "How can we overload but not overload too much that would just push me to that point?"

The need for coaches to effectively understand athletes’ impairments was a challenge intertwined with effective training strategies. As A1 highlighted: “I feel like there needs to be greater education around neurological disease, not for my sort of specific situation.” But also, how this impairment is impacting the individual, specifically, thus focusing on the interaction of the individual, their impairment, and their tendencies, with A1 expanding on this point: “If I’m not feeling right. I’m not soft. I actually hate taking any shortcuts in training because I have got that about wanting to make sure everything is to the second, perfect.” Cregan and colleagues [57] emphasized the importance of understanding athletes’ impairment in parallel to their knowledge of training and competition to create a suitable environment for athletes to perform at their best.

There are numerous sport-specific challenges athletes and coaches deal with including the need to travel for tournaments and training camps which comes at a cost financially, physically, and socially. There is also a clear emphasis on the athlete and coach dynamic to better understand athletes’ impairment, their abilities, and tendencies and how these factors interact, in order to better structure effective training and maximize athletes’ potential in the lead up into the Games.

#### Personal

The constant traveling for camps and tournaments, as well as daily training, has impacted participants’ personal lives as well, while they deal with challenges outside the sporting context. Some athletes relocated cities or countries for the opportunity to train with a coach, as A1 shared her experience moving in the recent years as they prepare for the lead up to Tokyo: “I’ve had to move recently. I’ve moved twice in … the last 14 months.” And they further expand on the impact of this in their personal life:

So, I’m not a natural-born full-time athlete. I’m too curious and I need to be stimulated intellectually as well as physically, and I need an outlet outside of this. I need to make a community outside of it as well. I think that’s really important. Having moved here, it’s hard to make a community anyway in [this city], I find, when you’re not working. And within the [sporting] community, I’m old, like within aggregate here, I’m one of the oldest. That’s difficult. I love [my teammates] and they’re my favorite people. But to have a peer would be nice.

The extensive commitment to their sport leaves athletes with limited opportunities to garner income outside of sport, leaving athletes with limited income during their competitive years. This point was highlighted by A2 who described how strategic he/she needed to be in order to save to survive until the Games:

Strategic in terms of work. So, this year, because it is so crazy leading up to Tokyo, I’ll end up not working for a good four months in total. So, to actually survive, I need to… obviously, that side of things I need to be quite smart about, which can be hard.

There is a need to focus on athletes’ health and the consequences of the increased time devoted to preparation for training, competition, and extensive traveling which takes away from other aspects of athletes’ lives [58]. Coaches also deal with extensive time away from family, due to travel for tournaments and training camps. However, having a family that is prepared for these challenges and can anticipate the changes leading up to the Games can be of great help, as alluded to by C4:

My wife knew [demands] would ramp up. And yes, it is ramping up with more camps, more travel, more commitment, more time I guess, or mental energy invested. So yes, there is still challenges, especially from my position where my family is based in [one State] and [I’m] spending sort of 50/50 between [two States].

Coaches also highlighted the importance of keeping themselves healthy mentally and physically in order to be able to be at their best during the Games to support their athletes. Being the year of the lead up to the Games, the pressure of performing at the Games has increased and with it, the psychological pressure for coaches has also increased, as highlighted by C2:

It’s a little bit of the pressure now, of performance. Previously, it’s great that you know that your athletes are there, et cetera. So, for me personally, I guess now, it’s starting to think a little bit about results, and how that affects me psychologically, and how that affects me psychologically in working with the athletes, as well.

Therefore, the lead up to the Games increased the demands for athletes and coaches’ time as there are increases in training camps and qualification tournaments. The increased time means sacrifices in other areas of participants’ lives. For coaches, it is mainly time away from family, while for athletes, they perceive this as time away from their social circles and potential education or vocation developments.

#### Uncertainties

The uncertainties mainly revolved around the sport, however; were different from challenges because they were unexpected events in the future that were decisive for athletes’ performances. One of the main concerns for all the participants was the outbreak of COVID-19 and its implications on sport-specific preparations for the Games, including upcoming qualifying tournaments and training camps. An athlete (A4) highlighted how they are approaching the uncertainty of whether the upcoming tournament will proceed:

Now that Northern Italy’s shut down because of this bloody coronavirus, that’s another challenge there. Who knows, who knows? You just keep going as if everything is going to be fine and just hope that it is, yeah.

In addition, there is equipment that had been ordered for the Tokyo2020 Games that is being manufactured in China, with factories shut down and back-order with shipments, there are concerns regarding whether the equipment will arrive in time for athletes to get accustomed to new equipment prior to the Games. One of the coaches, C3 explained how this could pan out for their athletes:

We’re getting our Tokyo 2020 [equipment]. We’re supposed to get them in April, but because of the virus, the factory in China is [currently shutdown] and shipment might be delayed. They’re the same brand of [equipment], but the geometry… some of the parts a little bit longer, other parts a little bit different. So, they have subtle differences that our athletes and coaches worry about.

There are also uncertainties regarding whether qualifying tournaments will occur and whether athletes will be able to qualify for the Games. Irrespective of COVID-19, some felt that the qualification tournaments and selection decisions for the Paralympic Games are made too close to the Games. This proves difficult for athletes on the cusp of qualification, as their family and loved ones will not be able to book travel until the selection is confirmed, and by that time, many places would be booked out and unavailable. This is additional stress added to athletes’ preparation as the Games approaches. Interestingly, none of the participants anticipated COVID-19 to become a pandemic and at the time of the data collection, the uncertainty was more geared towards whether the flu would impact their preparation for the Games. This demonstrates how these uncertainties could influence or shape athletes’ and coaches’ experiences and the inability to anticipate all outcomes. While participants reiterated the importance of just continuing day-to-day training until given a reason not to, follow-up studies exploring the impact of circumstances such as COVID-19 and how a pandemic impacts or creates new challenges for athletes and coaches is vital.

Other uncertainties regarding the Games pertained to competition days. Currently, there are uncertainties regarding how many staff will receive accreditation, resulting in challenges for coaches on how to prepare athletes’ competition strategies. C3 explains how this could be an anxiety-induced situation for coaches as they prepare their strategy for the Games:

There’s probably signs of some anxiety around some of the things and I think from a coaching perspective, I think our main source of anxiety is the day where we have the most chances to win gold. We have a lot of our athletes on one day and we only get a certain number of coaching accreditations. Can we service everybody to the level that the athletes deserve at the Paralympic Games?

Similarly, athletes’ concerns revolve around competition performance [35, 36, 38]. More specifically, how the environment (e.g., the reaction of the crowd) and the athlete (e.g., stress levels and more importantly, how their impairment may be on the day of) would feel, as alluded to by A3: “Hopefully we can do what we can. But, there’s always that uncertainty, or what if something happens, or one night of not as much sleep and then pushing yourself at competition.”

Collectively, the results highlight the depth and breadth of uncertainties athletes and coaches face in the lead up to a Paralympic Games. The main uncertainty is pertaining to the development of the novel coronavirus, COVID-19, which has impacted training camps, qualification tournaments, and delivery of equipment for Tokyo 2020. In addition, there are uncertainties regarding accreditation which has created a complex situation for coaches to navigate and prepare for as they attempt to develop a training environment similar to the Games. Last, there are concerns regarding athletes’ physical and mental state on event day, with some athletes concerned regarding how their body responds and whether there will be any impairment-related complications to deal with on the day of the competition. Generally, there are a wide range of factors that impact athletes’ ability to control outcomes within their environments and elite athletes have been known to appraise challenges and strategize solutions to cope with uncertainties to optimize their performance [59]. Here too, similar to Olympians, the Paralympic athletes were able to implement strategies to address some of the uncertainties.

### Strategies

Strategies highlighted a range of tools or systems participants have either implemented or plan to implement to reduce or remove some of the challenges. For both athletes and coaches, anticipation and preparation helped mitigate some of the uncertainties and remove miscommunication within the group. It also helped better prepare for Game strategies and they perceived that this, in turn, impacted their mental and physical health. Coaches also mentioned the need to manage both their personal and others’ expectations. While strategies differed, the objective was to ensure everyone was on the same page to further eliminate any miscommunication or misunderstanding.

#### Anticipation and preparation

Participants were cognizant of the variability that remains within training and competition (i.e., limited structure and continuous evolvement of the environment). Thus, to control some of the uncertainty, detailed planning regarding training, and competition has been designed to remove some of the current challenges regarding uncertainties leading up to the Games. Working in tandem, athletes and coaches co-create (see [60]) programs focusing on preparation both leading into, and at the Games. This mental preparation allows participants to be prepared for the unexpected by incorporating (and simulating) contingency plans that continue to evolve. As C1 highlighted, there are strategies in place to provide athletes independence in their decision making:

Wait and see, but I think communication with the athletes and then developing a plan where they can be self-sufficient as well. Or running scenarios, and having the athlete troubleshoot their way out of the situation, so they are independent.

Another coach, C2, explained the importance of preparation and planning as the difference between a successful and unsuccessful program as he/she highlighted a busy competition day scheduled for the Games and the consequences that hinge on it:

In these few hours is going to really be the difference between a successful program or not successful program, these matches. So, just then honing in on, "Okay, so what do we need? How do we need to be feeling at that time, on that day?"

Burns and colleagues [61] highlighted the importance of athletes’ meticulous planning and preparation, and reliance on routine to face uncertainties associated with Games day performance and environment. Athletes have also utilized visualization and imagery strategies to mentally prepare for upcoming events [61] and the athletes and coaches in this study mentioned using previous Games experience to develop strategies for the upcoming Games. This allows participants to better contextualize and create realistic expectations that will allow them to better prepare. A2 expanded how they use their experience from previous Games to remain confident and prepare for upcoming Games:

Yeah, definitely, because sometimes you just don’t expect some things that can happen. Yeah. So, I think just having gone through it before and played even just other tournaments, it didn’t have to be such a major one, but you sort of learn little things along the way.

The participants also noted preparation and planning helped reduce the physical and psychological stress and the importance of finding better ways to document and share information to avoid assumptions [61, 62]. Coaches also mentioned incorporating squad bonding opportunities during camps to increase chemistry between the team. Coaches used various Games during training to increase the chemistry between the squad including both sport- and non-sport-related activities (trivia, benchmark challenges, etc.). In addition, they simulated competition environment in training and presented various constraints (potential or expected delays, interruptions, missing equipment/gear) to allow athletes to get accustomed to the competition challenges and prepare to deal with uncertainties working together with their teammates. Therefore, while some challenges may differ between Olympic and Paralympic athletes (e.g., impairment-related nuances), the approach and strategies utilize are similar between the groups (i.e., anticipate and prepare).

#### Managing expectations

Coaches highlighted the importance of managing expectations. These included expectations of themselves, as many coaches are too hard on themselves and at times unrealistic. C3 explained their expectations for themselves which was echoed across the interviews with other coaches: “I’ve probably set higher expectations on myself than what I set on the athletes.” It is important coaches realize that not everything is within their control and there is a need to welcome a degree of uncertainty and variability so they can manage unexpected events or outcomes. Further, coaches stressed the importance of managing others’ expectations. This included their support staff and ensuring that the support staff understands their roles, and expectations of one another, removing any miscommunication that may occur during a highly intense and fast environment. As C1 explained:

But I think when you’re on the road it’s my expectation that everyone should be 24/7 job but not everyone treats it that way I don’t think. Yes, you do need downtime and stuff but there’s a job to be done and it’s got to be done. And that probably comes back from me being an athlete and also my old job that was a 24/7 sort of workshop that had deadlines to be made.

Confidence and understanding of each other’s roles and abilities is also vital so that the process is smooth. Athletes echoed a similar sentiment, in expecting their support staff and coaches to be at their best while having confidence that this will occur, as explained by A3:

So right now, they’re really, really good. And it’s been good with being able to work on a few things with the support staff around competition or on the day. So just that we’re all on the same page of what I need and what I need after my event and how to execute all those. All the water and the protein and the nutrition and wanting to get on with the race and just all be on the same page about what time I have my race at the start line and that. Which is already really good.

Considering they were more than six months away from the Games, this became a strategy to simulate and tweak athletes’ perceptions and preparations, and ultimately align their experience with their expectations. In addition, coaches also highlighted the importance of managing athletes’ expectations and what the athlete should expect of their coaches and support staff. This strategy helps reduce miscommunication and sets realistic expectations on how much support the athlete receives. The athlete can prepare their training and approach according to the boundaries established and this was echoed by coaches, including C3:

I think some systems need to go in place just to manage expectations. I think we need to be firm enough to set them, especially in the competition environment. So, that’s maybe as a coach or a group we need to [establish]. This is what you’re going to expect from us.

For athletes, on the other hand, it is important to ensure that they avoid grand expectations without accepting potential shortcomings (i.e., falling short of their medal goal). Thus, coaches have chosen to focus on the process over outcome, reiterating the importance of commitment to training and the strategies, a positive outcome would only be a bonus. Given the pressure of medal targets from senior management, consistent messaging becomes critical to ensure coaches focus on the right word selections and emphasizing the process over outcome, as highlighted by C4:

Certain things happen. People get sick, people get injured. But I think it’s about being real, or realistic. And I think whether that’s the humbling and not focusing on the outcome but focusing on the processes.

Another coach, C2, highlighted that while the expectation is gold medals, how athletes approach the Games will be important and the focus should be on the process and not the outcome:

I think the expectations are a bit narrower. I mean, now, there’s expectations of medals. And so, I think how we manage it, is just putting the processes of what we need to do, week to week now, day to day, hour to hour, once we get to the Games.

In addition, coaches were cognizant of having extra support ready for any athlete that did not achieve their expected goals, as the disappointing result could be a challenge for athletes to overcome. Coaches are counting on the wider Australian Paralympic Team to support in case of crises like this, but are aware that they have to be looking for the initial first signs of distress before seeking help from other staff, as alluded to by C5:

If there are athletes who may not [have] perform[ed] as well as what they hoped, PA, they had got so much support over there, we’ll tap in. If I feel like there’s some issues around that, then I’ll call on the PA specialists to come in and because they’ll have psych [support] and I’ll have one there just to help with that sort of thing.

Thus, athletes and coaches appear to put too much expectation on themselves, while coaches work to shift athletes’ expectations to the process and not the outcome. It is clear that all the participants expect the entire staff to work diligently during the Games, with everyone having a clear understanding of their roles and athletes’ needs. Sports also have strategies in place to manage athletes’ expectations during disappointing results at the Games.

### Post-Tokyo

All the participants were focused on their current goal of achieving the best they can at the Tokyo 2020 Games, thus, conversations regarding post-Tokyo were a mere reflection of what the athletes and coaches miss and what things they would be looking to incorporate into their lives with the reduced workload post-Games. For athletes and coaches, inevitably, the performance at the Games dictates certain outcomes post-Games. Thus, there was the theme of ‘Pressure.’ Personally, athletes and coaches were looking for time off after the Games to enjoy their social life, spend time with family and friends, and resume their hobbies or occupation/education that had been put on hold. However, coaches were also aware of the advance planning that goes into the program from now, as they would need to prepare, plan, and book travels for tournaments post-Games, thus, planning never stops in a cycle.

#### Pressure

Both athletes and coaches understand the importance of performance at the Games. Most importantly is that given all the time they have invested, they would like to capitalize on this with a positive performance outcome. However, coaches highlight any unsatisfactory outcome at the Games could cost them their jobs but were fine with this and accepted it to be the nature of their position in sport. Thus, even though having a guaranteed contract post-Games would help relieve some pressure, lack of it did not increase their stress levels, nor does it stop them from wanting their athletes to perform at their best.

From the athletes’ point of view, some saw the performance at the Games as opportunities for additional income revenue as medals and world stage accolades may help garner sponsorships and public speaking, as explained by A2:

After such [a] major Games and, if there’s a good result, some fun things do come out of it. But again, I’m just sort of focusing on Tokyo and then those additional things are all bonuses and really that come out of a good performance. So then financially, I guess, that opens up more doors for me. So then if I do put aside several months to train and not work, that’s where a big benefit comes in for me, post-Games.

Others are in a grey area in their careers and thus, a poor performance may put retirement at the front of their thinking. Some athletes, however, look at the Games as an opportunity to (re)establish themselves and look forward to 2024 as either a revenge season or protecting their crown. As highlighted by A1: “My plan has always been to win and then defend, and if I lose then it’s like, "let’s go and reclaim”. Therefore, while athletes and coaches understand the repercussions of their performance at the Games, they are solely focused on a good performance, rather than the consequences that may occur because of it. Recent literature suggests this to be an effective and positive approach to dealing with the pressure of the Games, as this type of approach by the coaches offsets the social pressure of the performance. In turn, this increases athlete motivation and engagement which have been previously associated with higher medal counts in the Paralympic Games [63].

#### Personal

Participants highlighted that the preparation for the Games has been exhausting, leaving them disconnected from their families and social circles. In addition, extensive training has had a toll on their bodies. Thus, they are looking forward to a mental and physical break while incorporating their social life back into their lives. A5 highlighted, there is a desire to travel and put their sport on pause, briefly, as they have devoted two full cycles to prepare for the Games:

I haven’t really [thought about it]. After such a big training block. Years of [my sport]. I probably deserve a little bit of a break. I like to play tennis. So, I’d probably go play tennis in the summer and have a break. I love traveling. It would be good to have a break.

Some athletes also have put their education/vocation on the side as the build-up to the Games has neared, thus, are looking forward to continuing their career path once the cycle has ended. This is a common practice as high-performance athletes focus on maximizing their current potential by reducing or removing all distractors that can consume time away from the sport [64]. Coaches are very much looking for the time off to reload their energy, but also are cognizant that the planning for the next cycle has already commenced. However, they also realize their health will be focal to the success of the program and a systematic break would be necessary because their personality would not allow them to stop working voluntarily. The laser focus on the performance at Games comes at a cost of limited plans post-Games. As C4 highlights, there is a wide range of emotions that participants can experience at and post-Games which comes with the consequence of withdrawal, but the time off is necessary both physically and psychologically for the athletes and coaches:

So, you probably go through a whole ball of emotions post [Games]. And then you sort of come to a realization, "Okay, I’m all right now I can keep going." And so, it’s just being mindful of that, but we all need that time off.

Thus, it is important to prepare athletes and coaches to expect the rollercoaster of emotions that are experienced both during and post-Games. The inevitable ‘high’ of the Games will wane and be met with a ‘low’ which can be extremely difficult to deal with if the results at the Games do not meet their personal expectation [65]. A specific initiative (i.e., educational component) to inform and prepare athletes of the potential experiences and readily accessible resources (e.g., alternative career options, careers within sport, volunteering/mentoring opportunities, etc.) to deal with such circumstances will be vital for athlete and coach well-being.

### Practical implication

The findings of this case study have several practical implications. First, results highlight the unique challenges athletes and coaches face and this understanding could help stakeholders (i.e., directors, coaches, athletes) better prepare to face these issues in the future. A platform to facilitate a more effective planning and communication across the organization can alleviate some of these challenges, as noted by the participants in this study and it would also create a space to communicate expectations for one another and establish clear boundaries for coaching staff and athletes. The lack of communication, mis- or lack of understanding was at the forefront of some of the challenges both athletes and coaches faced. Sport organizations can incorporate strategies within the system to mitigate some of the challenges by providing resources (e.g., creating a communication platform, impairment-specific resources) where additional support is most needed and allocating an appropriate budget to support interventions (e.g., additional support staff, post-Games support). For instance, our qualitative data suggest a need to support coaches by providing additional resources during the last year of the Quadrennial (i.e., additional staff and extended budget). The platform can be utilized to create resources for coaches and staff on key impairment-related factors specific to each athlete with details pertaining to athletes’ needs within their daily training environments, traveling long distances, the physical and mental state leading up to and on the day of the competition. This would further facilitate effective communication and trust between the coaching staff and athletes and reduce the chances of miscommunication. The additional resources can also allow coaches to select appropriate tournaments and camps, find suitable accommodations, and identify and invest in equipment and technology to enhance athlete experience and team communication. However, it is important to consider the implications of this for the pathway to ensure the increase of resources (i.e., budget, staff) at the high-performance level is not an (in)direct hindrance to the rest of the pathway (i.e., reduced resources elsewhere which could have long-term implications for the sport). Furthermore, the addition of support staff could reduce coaches’ workload by mediating coach-athlete interactions to increase the quality of communication and opportunities for engagement. In turn, this could provide athletes with a specific person to address the subtle and nuanced topics associated with their day to day training and/or impairment related factors. Although, considering one of the main challenges was the impact of planning and communication, introducing a new staff member towards the end of the Quadrennial should be done with great caution and consideration. Recognizing and acknowledging the unique challenges Paralympic athletes face as they prepare for a major Games is important to improving access to relevant resources and support.

It is also important for sporting organizations to support coaches during the lead up to the Games and ensure their self-imposed high expectations are manageable and negative results do not impact the coaches’ psychological and physical well-being. In addition, as alluded to in previous literature [64, 65], having a system in place to support athletes and coaches post-Games is vital. First, participants have spent extensive amounts of time in their current environments preparing for the Games with many sacrifices in their personal lives. Second, undoubtedly, they will be experience intense feelings of either euphoria (e.g., meeting/exceeding expectations) or sadness (e.g., not meeting medal targets) pending on the Games’ outcome. Last, while they return home, they are less likely to be spending as much time in environments they are accustomed to pre-Games (e.g., training facilities, rehabilitation and recovery centers, interaction with coaches and teammates, etc.). Therefore, it is beneficial to develop a support mechanism to oversee athletes and coaches’ adjustments post-Games.

## Conclusion

The authors’ understanding of the challenges and strategies of Paralympic athletes and coaches in lead up to the major Games is limited. The findings of this study captured the experience of Australian Paralympic athletes and coaches in preparation leading up to the Paralympic Tokyo Games. There was a wide range of challenges noted with participants using a range of strategies to mitigate or anticipate any upcoming obstacles. More specifically, there are key challenges pertaining specifically to the sporting environment (i.e., budgetary constraints, communication, personnel management) and individuals’ lives (i.e., social, vocation/education, residency) along with uncertainties (i.e., accreditation, qualification, COVID-19) that are intertwined withing the sporting context and society in general. Participants mitigate some of these challenges by focusing on effective communication and preparation of contingency plans. An obvious shortcoming of this study was that data collection occurred pre-pandemic, when the Tokyo Games were still scheduled to occur in the summer of 2020. While the findings shed light on customary things that occur in lead up to any major Games, a tailored study examining athletes’ experiences post-pandemic (e.g., if the Games are rescheduled from 2020 to 2021, or canceled in its entirety) could be vital to inform of how policymakers and sport organizations can better prepare for future unpredicted events. Interestingly, some of the previously reported challenges for athletes and coaches in the Paralympic setting were not evident in this study (e.g., challenges to travelling with an impairment, access to facilities, etc.). This could be because these athletes and coaches are performing at the highest level, and focused on overcoming sport-related challenges, and less so on impairment-related barriers [57]. Both Dieffenbach and Statler [66] and Macdougall and colleagues [1] suggest Paralympic athletes’ needs at the high-level are different than those at lower levels, suggesting athletes may become accustomed to high-performance needs, accept existing challenges as part of their daily routine, and identify ways to mitigate barriers while exploring ways to optimize their environment to perform at their best. The findings of this study highlight some of the systematic challenges Paralympic athletes and coaches likely face leading up to any major event and can help us better understand the capacity and resources necessary to provide targeted support leading up to the Games.
